# Extreme wettability of nanostructured glass fabricated by non-lithographic, anisotropic etching

**DOI:** 10.1038/srep09362

**Published:** 2015-03-20

**Authors:** Eusun Yu, Seul-Cham Kim, Heon Ju Lee, Kyu Hwan Oh, Myoung-Woon Moon

**Affiliations:** 1 Institute of Multidisciplinary Convergence of Matter, Korea Institute of Science and Technology, Seoul 136-791, Republic of Korea; 2 Department of Materials Science and Engineering, Seoul National University, Seoul 151-742, Republic of Korea

## Abstract

Functional glass surfaces with the properties of superhydrophobicity/or superhydrohydrophilicity, anti-condensation or low reflectance require nano- or micro-scale roughness, which is difficult to fabricate directly on glass surfaces. Here, we report a novel non-lithographic method for the fabrication of nanostructures on glass; this method introduces a sacrificial SiO_2_ layer for anisotropic plasma etching. The first step was to form nanopillars on SiO_2_ layer-coated glass by using preferential CF_4_ plasma etching. With continuous plasma etching, the SiO_2_ pillars become etch-resistant masks on the glass; thus, the glass regions covered by the SiO_2_ pillars are etched slowly, and the regions with no SiO_2_ pillars are etched rapidly, resulting in nanopatterned glass. The glass surface that is etched with CF_4_ plasma becomes superhydrophilic because of its high surface energy, as well as its nano-scale roughness and high aspect ratio. Upon applying a subsequent hydrophobic coating to the nanostructured glass, a superhydrophobic surface was achieved. The light transmission of the glass was relatively unaffected by the nanostructures, whereas the reflectance was significantly reduced by the increase in nanopattern roughness on the glass.

Functional glass with superhydrophobicity/hydrophilicity, anti-condensation or low-reflectance has been developed for various applications, including windows for automobiles, vehicles or ships; inner or outer buildings windows; and display panels for electronic and medical devices and other optical equipment^1^,^2^,^3^. Functional glass surfaces require nano- or micro-scale roughness, which is difficult to fabricate directly on glass surfaces^4^,^5^. Hydrophobic and superhydrophobic surfaces are obtained by applying coatings of a low-surface-energy material to nanostructured surfaces; these coated surfaces can mimic micro-, nano- or hybrid structures, such as that of the lotus leaf, which has a high water contact angle (CA) and low contact angle hysteresis (CAH)^3^. Conversely, superhydrophilic glass with extreme wettability has been developed for anti-fogging, self-cleaning, and water collecting surfaces^6^,^7^. Superhydrophilicity is improved by the presence of surface nanostructures coated with high-surface-energy materials^8^. Therefore, surface coatings on glass surfaces are required to both control the surface energy and introduce extreme wettability.

Recently, several studies have focused on the fabrication of regular nano- or microscale patterns on glass to improve its wettability or anti-reflectance. Some researchers have reported a fabrication method for introducing surface roughness onto glass by using a self-mask pattern and a thin metal layer such as used in the Ni dewetting method. Using the inductively coupled plasma etching method, nanostructured glass with a high aspect ratio has been fabricated for use on solar panels. However, this method requires an additional step for removing the metallic mask with acidic chemicals^9^,^10^. Zeze et al. reported a lithography-free method for the fabrication of high aspect ratio quartz columns, by using reactive ion etching or chemical etching with a CF_4_ and Ar gas mixture^11^. They suggested that the preferential etching to form columnar structures is due to the presence of localized metallic impurities in the quartz matrix and to the non-uniform distribution of the electrostatic fields at the rounded edges of the contact mask. However, the suggested methods are limited by the low density of the columnar structures on the SiO_2_-based quartz-type glass. It has been reported that in contrast to quartz or Si formation using volatile compounds such as SiF_x_, the surface roughness of soda-lime glass would not be affected by reactive ion etching with CF_4_ or SF_6_ plasma treatment because of the presence of nonvolatile oxide components such as CaO and Na_2_O^12^. It has also been suggested that because of the production of nonvolatile products (such as NaF or AlF_3_) during etching with halogen gases (such as CF_4_ or SF_6_), glass surfaces containing Na or Al would be protected from etching^4^. The etch rate of soda-lime glass by CF_4_/CHF_3_ plasma, 4 nm/min, is much lower than that of glass that does not contain Ca or Na, 45 nm/min, under the same processing conditions^12^. It is a challenge that glasses containing nonvolatile elements form surface nanostructures under a plasma-based anisotropic etching method. Owing to the difficulty of directly forming nanostructures on general glass, researchers have suggested the introduction of an additional layer on the glass via spraying nano- or microscale particles^13^ or via sol-gel coating with a porous thin film. However, the low interfacial adhesion strength between the glass and the additional layer presents a limitation to the widespread application of this technique^14^.

In this work, we provide a novel non-lithographic method for fabricating nanostructures directly on glass via the introduction of a sacrificial SiO_2_ layer and subsequent anisotropic plasma etching with reactive plasma. The SiO_2_ layer was deposited onto glass as a sacrificial layer that was subsequently CF_4_ plasma-treated to form a nanostructure by using a well-known anisotropic plasma etching technique (Figure 1a)^15^. In turn, SiO_2_ nanoscale pillars acted as surfaces that resisted the continuous plasma etching (Figure 1b), resulting in nanostructure formation on the glass surfaces. The glass regions covered with SiO_2_ pillars are etched slowly, whereas the regions with no SiO_2_ pillars are etched rapidly, resulting in the formation of nanopatterns on the glass. Our process requires post-processing via water hydrolysis to remove residual metal fluoride and to enhance the nanopattern roughness. With CF_4_ plasma treatment alone, the roughness caused the nanostructured glass to become superhydrophilic^16^. Subsequently, a low-surface-energy material was coated on the nanostructured surfaces to render them superhydrophobic (Figure 1c)^17^.

A surface analysis of the plasma-treated surfaces was conducted. Water CA and CAH measurements were taken, and water condensation experiments were performed to characterize the effects of the surface treatments on the wettability and nucleation/growth of water droplets, respectively. Changes in optical transmittance were also investigated by using UV-vis spectroscopy to estimate the effects of nanostructure formation, under various plasma durations, on the anti-reflectance of the glass substrates.

## Results

### Morphology and microstructure

Soda-lime glass surfaces were etched with CF_4_ plasma for varied durations, as shown in Figure 2a–c. No noticeable surface roughness was observed on the bare glass after up to 60 min of plasma etching. Using AFM, it was found that the roughness was slightly increased, from 0.095 nm for pristine glass to 1.849 nm after 60 min of CF_4_ plasma etching. Glass surfaces can be uniformly etched with reactive plasma ions because they contain Ca and Na, which protects them from being etched by the plasma-based anisotropic etching mechanism^4^,^12^. For glass coated with a 1-μm-thick sacrificial layer of SiO_2_, the nanostructures on the sacrificial layer evolved into pillar shapes with a high aspect-ratio, 6.6:1. A side view of these pillars can be observed in Figure 2d–e, where the SiO_2_ layer is brighter than that of the glass substrate in the SEM image. With continuous CF_4_ plasma etching, the SiO_2_ nanopillars can serve as etching masks for the selective etching of the glass substrates, resulting in the formation of nanostructures on the glass, as shown in Figure 2f. Here, the SiO_2_ pillars were etched away, providing a glass surface with hierarchical nanostructures featuring 500-nm-wide bumps decorated with 15- to 30-nm-wide pillars. The surface roughness was measured on the glass with and without the SiO_2_ sacrificial layer following varied CF_4_ plasma etching durations, as shown in Figure 2g. By estimating the width, *d*, and the height, *h*, of a nanostructure, as well as the distance between nearest nanostructures, *s*, an equation for the dimensionless parameter of roughness, *r*, was adopted from Ref. 18: *r = * 1 *+ πdh/(d + s)*^2^, where the minimum roughness of a flat surface is 1. Based on the measured geometries shown in Figure S1, the roughness of the pristine glass was measured as 1.001 and that of the plasma treated glass without nanopillars was below 1.01. In the case of the nanostructured glass, the roughness was measured to be greater than 3, which is significantly greater than that of the glass without the SiO_2_ layer.

### Wetting properties

With increased plasma treatment duration, the surface morphologies changed, which affected the wetting behavior, as shown in Figure 3. The water CA and CAH were measured on nanostructured glass before and after the application of a hydrophobic coating. The glass without the SiO_2_ layer has no significant surface features; therefore, the CA before and after CF_4_ plasma etching was almost unchanged, at approximately 30°. The CA of glass without the SiO_2_ layer increased to 90° with the hydrophobic coating, which is similar to the value for the flat surfaces coated with plasma-polymerized hexamethyldisiloxane (pp-HMDSO), as shown in Figure 3a and b. In the case of the nanostructured glass, the surface wettability was significantly improved by a combination of the high surface energy of the plasma treated glass or SiO_2_ surface and its nanoscale roughness, resulting in a large reduction of the CA to less than 5° after 5 min of plasma treatment. With the hydrophobic coating, superhydrophobic surfaces were achieved after 15 min of CF_4_ plasma treatment, and the CAH was measured to be as low as 3°. As shown in Figure 3c, the long-term durability of the superhydrophobic and superhydrophilic properties was assessed over 50 days and was found to be almost unchanged for both properties because of the nanostructure effect^10^,^19^.

The superhydrophobic and superhydrophilic nanostructured glasses were tested for anti-condensation with a simple water spraying test under ambient air conditions and with a temperature change from −17 to 25°C under supersaturation conditions, as shown in Figure 4. As the micro-scale water droplets were sprayed onto the superhydrophobic lens of the eye glass, the water droplets showed high mobility with a very low CAH, resulting in dropwise condensation. Similar to the well-known lotus effect, the droplets all rolled off the superhydrophobic glass surface, as shown on the left side of the glasses in Figure 4a and Figures S2 and S3 in supplementary information. Conversely, liquid film formation was observed on the superhydrophilic surfaces, as shown on the right side of the glasses in Figure 4a and Figures S4 and S5. With the continued addition of water vapor, no water droplets were observed, and the transparency of the superhydrophilic surface was unaffected. This result was expected because it is known that superhydrophilic glass surfaces can be used for anti-fogging or anti-condensation applications under supersaturation conditions^20^,^21^.

## Discussion

EPMA analysis was performed to investigate the surface chemical compositions of bare and nanostructured soda-lime glasses after CF_4_ plasma etching, as shown in Figure 5a and b, respectively. In the case of bare glass, Fe and F were weakly detected (Figure 5a). Randomly oriented nanopatterns on the nanostructured glass were analyzed by using SEM in backscattered electron (BSE) mode, and the corresponding EPMA intensities for Fe and F components were measured, as shown in Figure 5c. The large increases in these two components on the nanostructured glass indicate the formation of metal fluoride, as shown in Figure 5b and c. The regions covered with metal fluoride are not easily etched, whereas the regions without metal fluoride can be rapidly etched through mechanical or chemical reaction etching. It should be noted that the unetchable metal fluoride on the nanostructures can be removed via a hydrolysis reaction in water^22^,^23^. Note that the conventional self-masking method by metal film dewetting requires post-processing with toxic acids to remove the metallic masks on top of the glass nanostructures^9^,^10^. Additional TEM observation on a 500 nm high/300 nm wide nanopillar (Figure 5d and e) revealed that Fe and F surrounded the nanopillar, whereas Ca and Si were in the body of the pillar, as shown in Figure 5f. From this result, it can be hypothesized that Fe, which originated from the chamber cathode upon the irradiation of F ions, would form the metal fluoride FeF_x_. Upon the formation of metal fluoride clusters on the SiO_2_-coated glass surfaces, the F ions may be etched by physical sputtering, whereas the surfaces that do not contain metal fluoride may be etched by both physical sputtering and chemical reaction mechanisms by forming SiF_x_-like volatile species^24^.

Water condensation (or anti-fogging) tests on glasses with different wettabilities were performed by increasing the air temperature from −17 (inside a freezer) to 25°C (in ambient air), as shown in Figure 4b. It was observed that the superhydrophobic surface was easily fogged with water vapor condensation as the temperature increased, whereas the superhydrophilic surfaces maintained their transparency, indicating that no water condensation occurred. It has been reported that the condensation behavior of superhydrophobic surfaces is related to the aspect ratio of the surface nanostructures. In the structures formed on the glasses in this study, the pillar height was not sufficient to resist dew formation; thus, water droplets of a few micrometers in size from a sprayed vapor may adhere to, rather than roll off, the glass surface at low temperatures, causing the glass surface to be blurred. Conversely, the superhydrophilic surface has sufficiently high surface energy to quickly form a thin water film, maintaining the glass surface transparency^25^.

Functional glass used in applications such as solar cell panels, automobile side mirrors or optical glasses for medical devices or surgery require anti-reflection properties as well as self-cleaning and anti-fogging properties. The optical anti-reflectance is related to the surface roughness and is an essential requirement for improving the optical efficiency of glass. In addition to increasing the hydrophobicity or hydrophilicity, controlled roughness plays a crucial role in reducing light reflectance by enhancing light scattering, which is similar to the moth-eye effect. Soda-lime glass without any plasma etching exhibits some incident light reflection and has a water CA of 30°, whereas the nanostructured glass surfaces show very low water wetting angles in the non-coated condition but a high wetting angle following pp-HMDSO coating, as shown in Figure 6a. Note that the nanostructured glass surfaces with or without hydrophobic pp-HMDSO coatings have very low light reflection and that a comparison of the transmittance spectra of the pristine glass before and after the hydrophobic coating process indicates that no significant decrease in transparency occurred as a result of the very thin layer (20 nm) of pp-HMDSO coating. Transmittance on the glasses without the SiO_2_ coating was slightly altered as the CF_4_ plasma etching duration was increased, as shown in Figure 6b. In the case of the nanostructured superhydrophobic glasses with etch durations of less than 30 min shown in Figure 6c, the surfaces remained transparent, whereas those with longer etch durations of 60 min had drastically decreased transmittance in the near-violet region due to the larger pillar size, as shown in Figure 2f. Compared with the pristine glass, the reflectance was not changed on the glasses without SiO_2_ coating, regardless of the plasma etching duration, as shown in Figure 6d, whereas a large reduction in the reflectance from 9 to 3 at the wavelength of 600 nm was observed on the superhydrophobic glasses shown in Figure 6e. Overall, the light transmission on the nanostructured glass surfaces was relatively unaffected by the nanostructures, whereas the reflectance was considerably reduced by longer CF_4_ plasma etching durations.

This simple and novel method to fabricate nanostructures for functional glass with superhydrophobicity/hydrophilicity, anti-condensation properties and low reflectance can be applied in various fields, such as for self-cleaning mirrors or windows in automobiles, vehicles and ships; windows inside and outside buildings; display panels for electronics; and other optical equipment for biomedical applications.

## Methods

**Figure 1:**
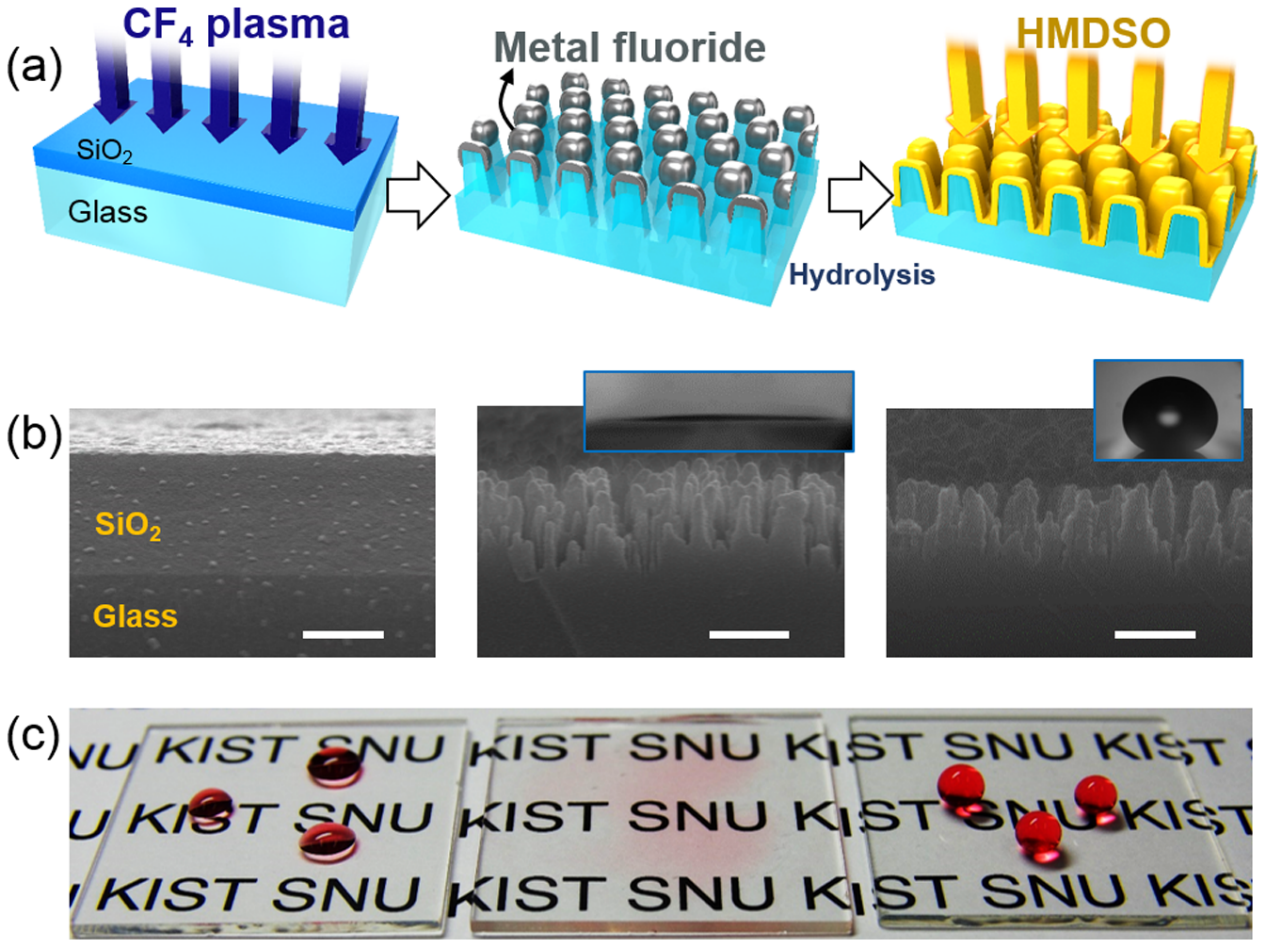
Schematic representation of the fabrication process of nanostructured glass. (a) Application of a sacrificial layer of SiO_2_ and reactive ion etching by CF_4_ plasma treatment. (b) Cross-sectional SEM images of SiO_2_-coated glass before nanostructuring (left) and CF_4_ plasma-etched glass with a SiO_2_ coating before (middle) and after (right) pp-HDMSO coating. Scale bar is 500 nm. (c) Optical images of pristine (left), superhydrophilic (middle), and superhydrophobic (right) glasses. Water was dyed red.

**Figure 2:**
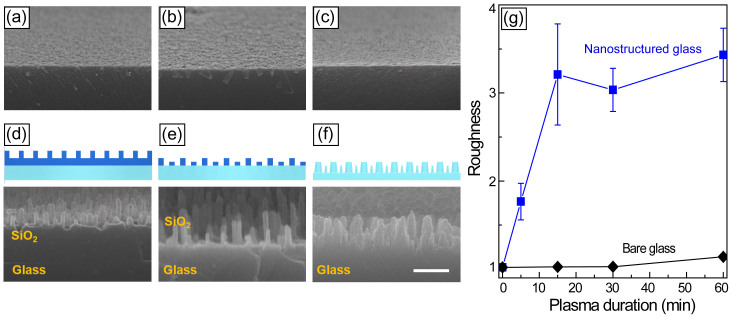
SEM images of CF_4_ plasma-etched glass surfaces and soda-lime glass cross-sections with varied CF_4_ plasma etching duration: (a) 15, (b) 30, and (c) 60 min. Schematic representation and SEM cross-sectional images of SiO_2_-coated glasses with varied CF_4_ plasma duration: (d) 15, (e) 30, and (f) 60 min. Scale bar is 500 nm. (g) Roughness measured on glasses with (blue) and without (black) SiO_2_ layer.

**Figure 3:**
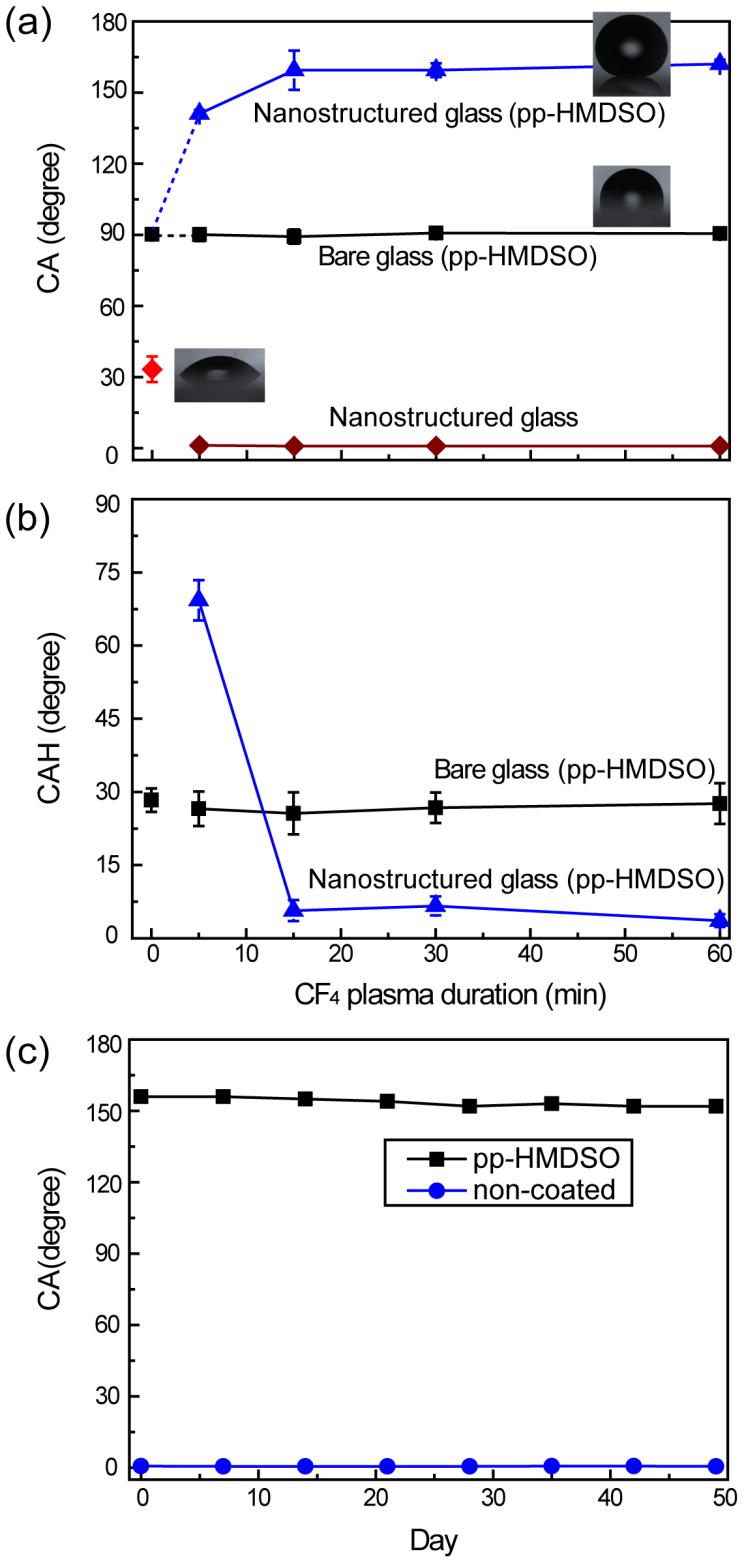
Measured wetting properties: (a) CA, (b) CAH, and (c) wetting durability of nanostructured glasses with and without pp-HMDSO coating.

**Figure 4:**
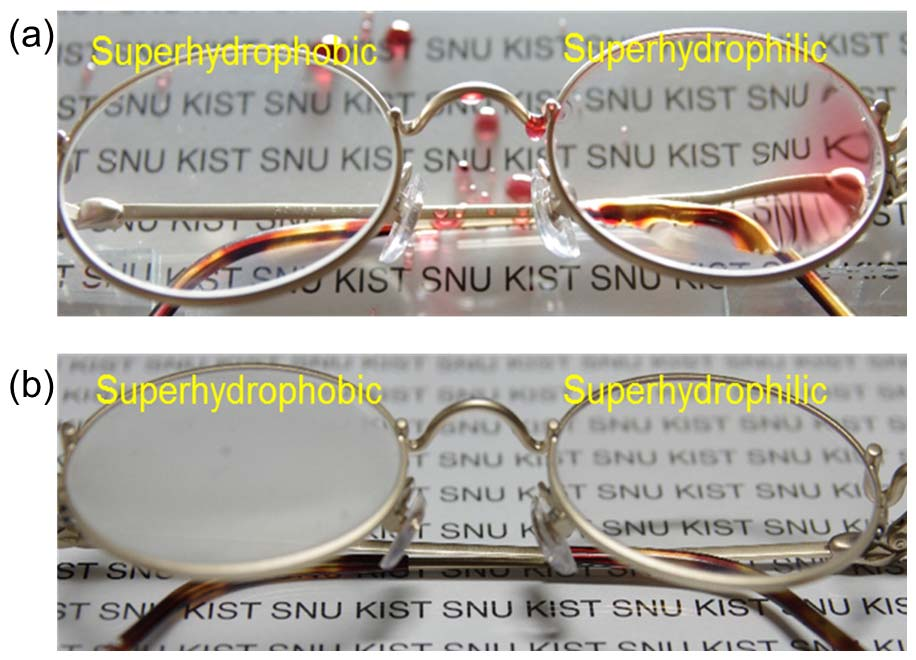
Superhydrophobic and superhydrophilic lenses in eyeglasses with (a) water spray test on glass surfaces and (b) the condensation test by an incident temperature increase from −17 to 25°C. CF_4_ plasma etching duration for both glasses was fixed at 30 min.

**Figure 5:**
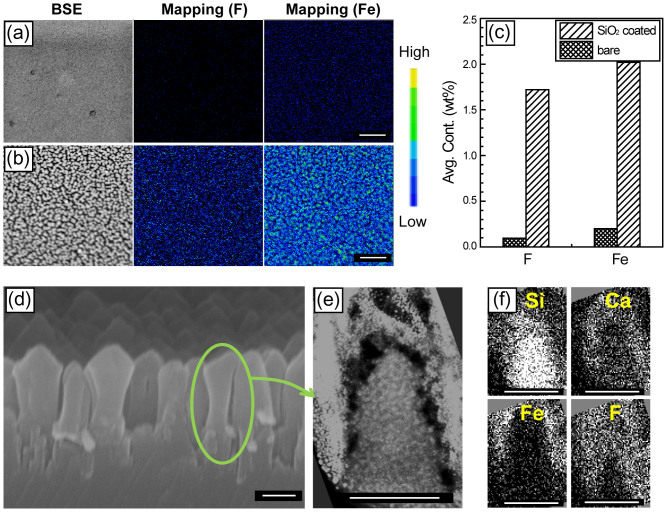
SEM image (left) and EPMA mapping (middle and right) analysis of glass surfaces for (a) SiO_2_-coated glass and (b) bare glass with F and Fe as the selected surface atoms. Scale bars are 1 μm. (c) EPMA intensity for Fe and F components on the samples in (a) and (b). (d) SEM image of nanostructured glass at an 80° tilt. Scale bar is 500 nm (e) TEM image of a single glass nanopillar and (f) its EELS area mapping of four components (Si, Ca, Fe, and F) where all components are false colored. (e) and (f) image scale bars are 300 nm

**Figure 6:**
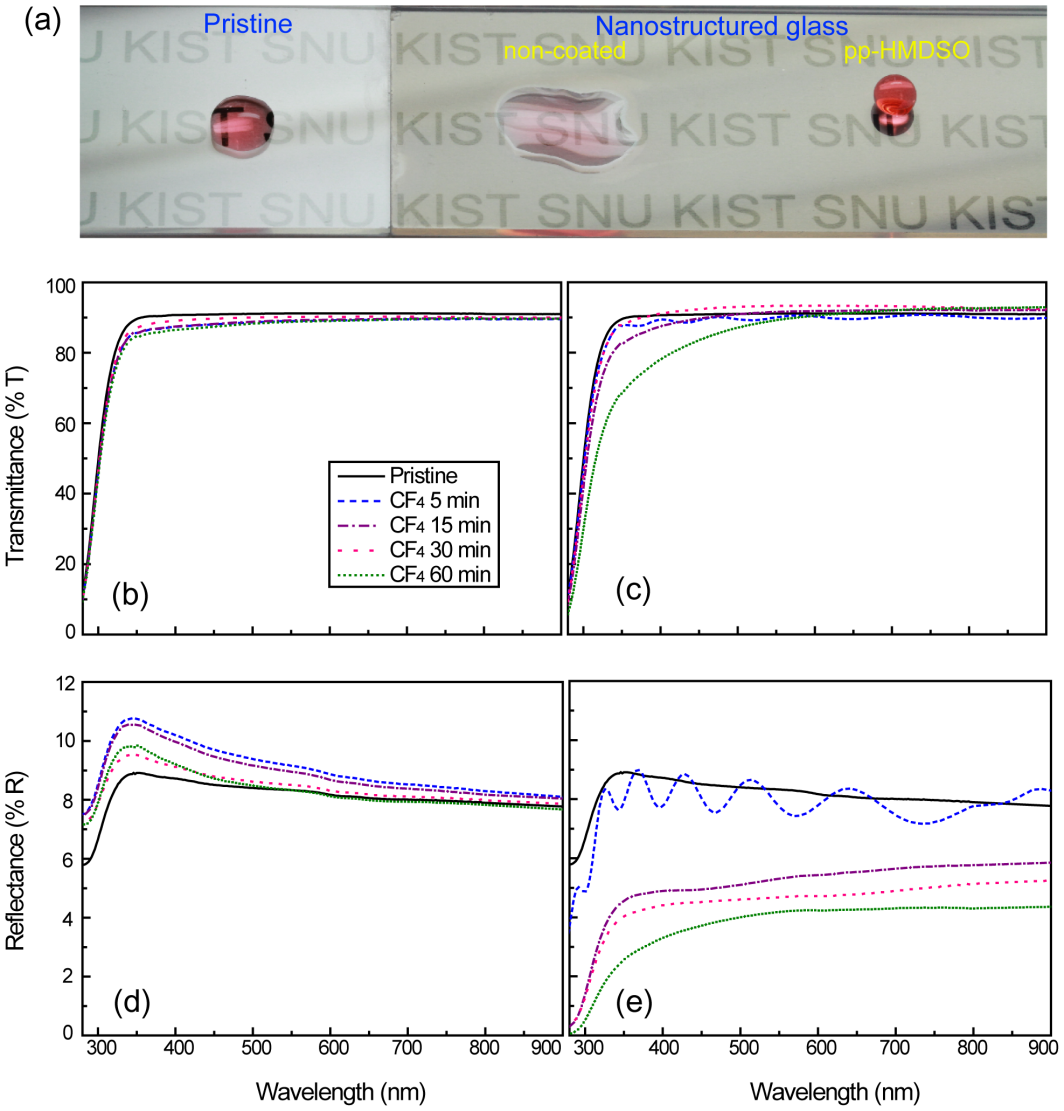
(a) Optical microscopic images of water droplets on three glass surfaces: pristine (left), superhydrophilic (middle) and superhydrophobic (right). Optical transmittance of the CF_4_ treated glass surfaces (b) without and (c) with the SiO_2_ coating. Optical reflectance (d) without and (e) with the SiO_2_ coating.

Soda–lime glass (Marienfeld, 1000412) with a thickness of 1 mm was utilized in the experiments. The glass surfaces were cleaned with acetone, methanol and 2-propanol. Then, a 1-μm-thick SiO_2_ overlayer was deposited onto the glass using plasma-enhanced chemical vapor deposition (PECVD) with a mixture of N_2_O gas and SiH_4_ gas^26^. After coating, the samples were etched using a glow discharge of CF_4_ gas with a treatment duration from 5 to 60 min by PECVD, as shown in Figure 1. The gas pressure and bias voltage were maintained at 30 mTorr and −600 V_b_, respectively.

After CF_4_ plasma etching, nanostructures were developed on the SiO_2_ layer-coated glass via the well-known method of preferential etching with a reactive ion. With continuous CF_4_ plasma etching, the SiO_2_ pillars became etching masks for the glass, resulting in nanopillars on the glass surfaces. The nanostructured glass was subjected to a hydrolysis process to remove metal fluorides from the surface, by using the water treatment method^27^. The glass surfaces were found to be superhydrophilic after nanostructuring. To reduce the surface energy of the nanostructured glass surfaces, a hydrophobic material was deposited onto the surface by the plasma polymerization of HMDSO (C:H:Si:O) using PECVD. The gas pressure was fixed at 10 mTorr, the bias voltage was −400 V_b_, and the deposition time was 15 sec^28^.

The wettability of the nanostructured glass surface was characterized by deionized (DI) water droplet CA and CAH measurements. For these measurements, water droplets of approximately 5 μL with radii of approximately 1 mm were gently deposited onto the surfaces using a micro-syringe. The advancing CA was measured by adding a sessile drop of DI water (~5 μL), and the receding CA was measured by removal of a sessile drop of DI water. The CAH was calculated as the difference between the advancing and receding CAs. All measurements were taken using a CA goniometer (Rame-Hart) in ambient air at 25°C with a relative humidity of 20–35%. The reported CAs were determined by averaging the measurements from five different locations on each sample. Glass with the two extreme wetting conditions, superhydrophobicity and superhydrophilicity, on each side was tested for water repellence and anti-fogging properties. Glass samples were stored in a freezer at −17°C for 60 min for the anti-condensation and anti-fogging tests, and then the condensation experiments were performed under ambient conditions.

The surface nanostructures were observed with a scanning electron microscope (SEM, NovaSEM, FEI), and their geometries were measured with an atomic force microscope (AFM, XE70, Park Systems). The surface atomic dispersion was measured with a field emission electron probe micro-analyzer (FE-EPMA, JXA-8500F, JEOL) and a transmission electron microscope (TEM, JEM-3000F, JEOL). UV-vis measurements were performed in the wavelength range of 250 to 900 nm using a spectrophotometer (Perkin-Elmer, Lambda 20) at room temperature. The spectra were recorded with air as the reference.

## Author Contributions

E.Y., K.H.O. and M.W.M. planned the study. E.Y., H.L., S.C.K. and K.H.O. performed material fabrication and some of the characterization steps. E.Y., S.C.K., H.L. and K.H.O. carried out the wetting-related experiments and TEM analysis. E.Y., S.C.K., K.H.O. and M.W.M analyzed the experimental data and results. E.Y., M.W.M. and K.H.O. wrote the manuscript. All authors discussed and commented on the manuscript and provided feedback.

## Supplementary Material

Supplementary Information Supplementary Information
