# Supplementary material for: Extreme wettability of nanostructured glass fabricated by non-lithographic, anisotropic etching
Source: Sci Rep. 2015 Mar 20;5:9362. doi: 10.1038/srep09362 (PMC4366763; doi:10.1038/srep09362)
Supplement: Supplementary Information [file srep09362-s1.doc]

**Supplementary Information**

**Extreme wettability of nanostructured glass fabricated by non-lithographic, anisotropic etching**

Eusun Yu^1,2^, Seul-Cham Kim^2^, Heon Ju Lee^1^, Kyu Hwan Oh^2^ & Myoung-Woon Moon,^1^*^,*^*

**^1^** Institute of Multidisciplinary Convergence of Matter, Korea Institute of Science and Technology, Seoul 136-791, Republic of Korea

**^2^** Department of Materials Science and Engineering, Seoul National University, Seoul 151-742, Republic of Korea

*To whom correspondence should be address: E-mail: [mwmoon@kist.re.kr](mailto:mwmoon@kist.re.kr)

**Geometrical details on the nanoscale pillars formed on nanostructured glass (SiO_2_-coated and CF_4_-treated)**


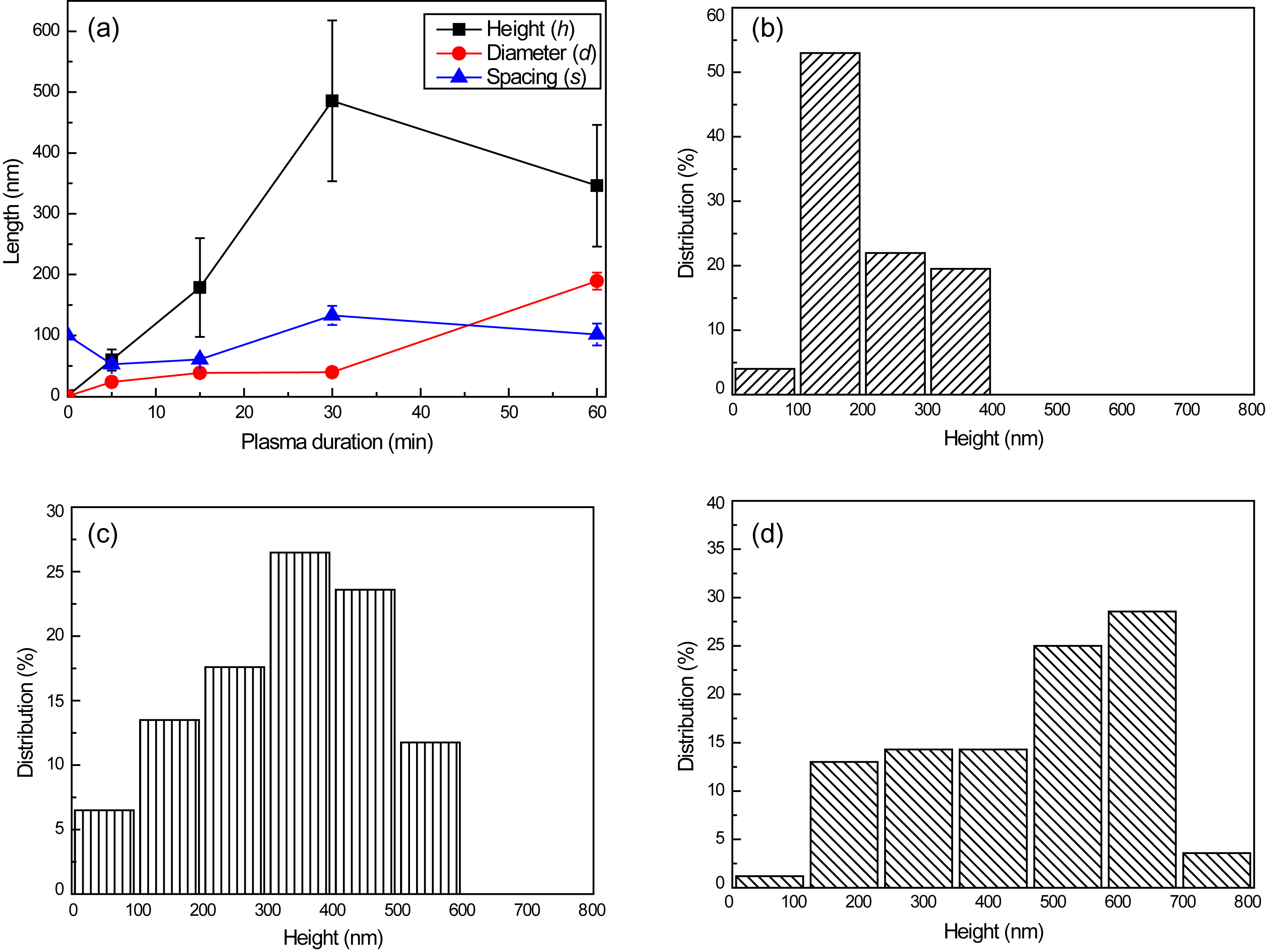


**Figure S1.** (a) Nanostructured pillar dimensions with respect to CF_4_ plasma etching duration; height, diameter, pillar spacing. Because of the large variation in the height of the pillars, the height distribution was measured for three different durations of CF_4_ plasma etching: (b) 15 min (c) 30 min (d) 60 min.

Anti-fogging test and water spray test on nanostructured glass with superhydrophobic and superhydrophilic treatments


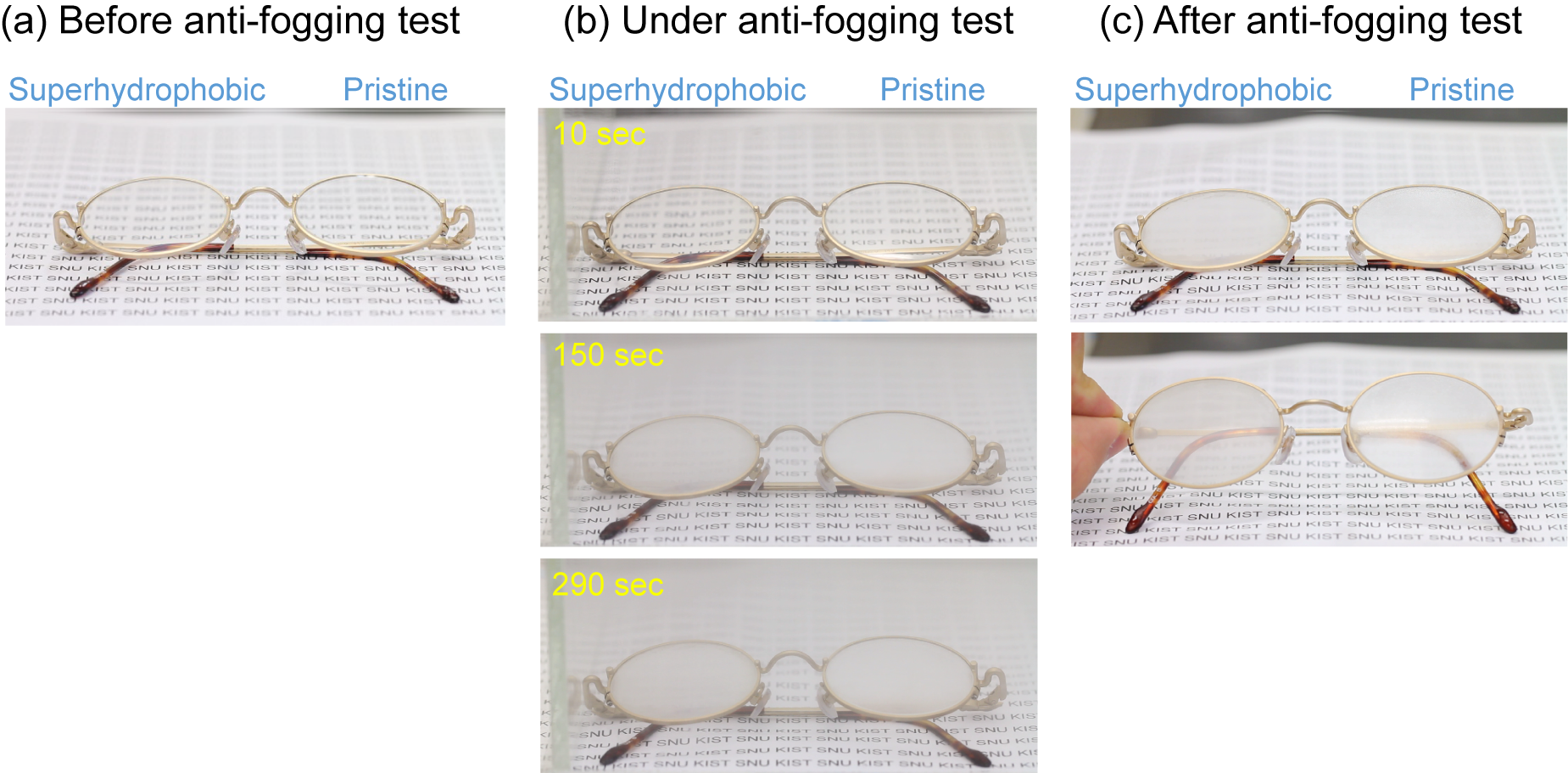


**Figure S2**. Optical images of superhydrophobic eye glasses under anti-fogging or condensation tests: the total test duration was 5 min under supersaturation conditions. The numbers in (b) are the test durations.


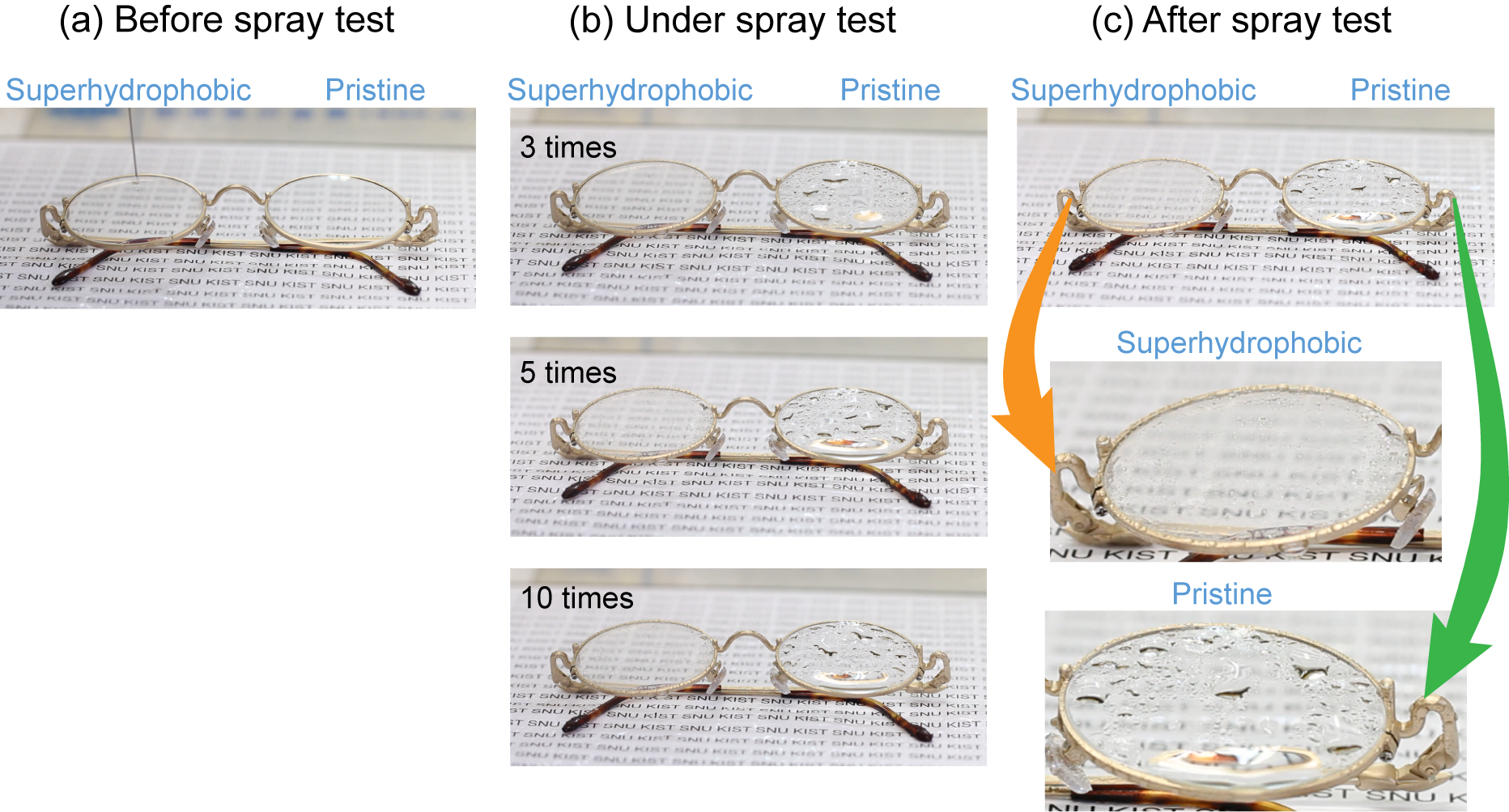


**Figure S3**. Optical images of superhydrophobic eye glasses taken (a) before, (b) under and (c) after spray testing under various surface conditions. The total number of spraying cycles is 10. Note that the water may roll off the superhydrophobic glass, as shown in (c).


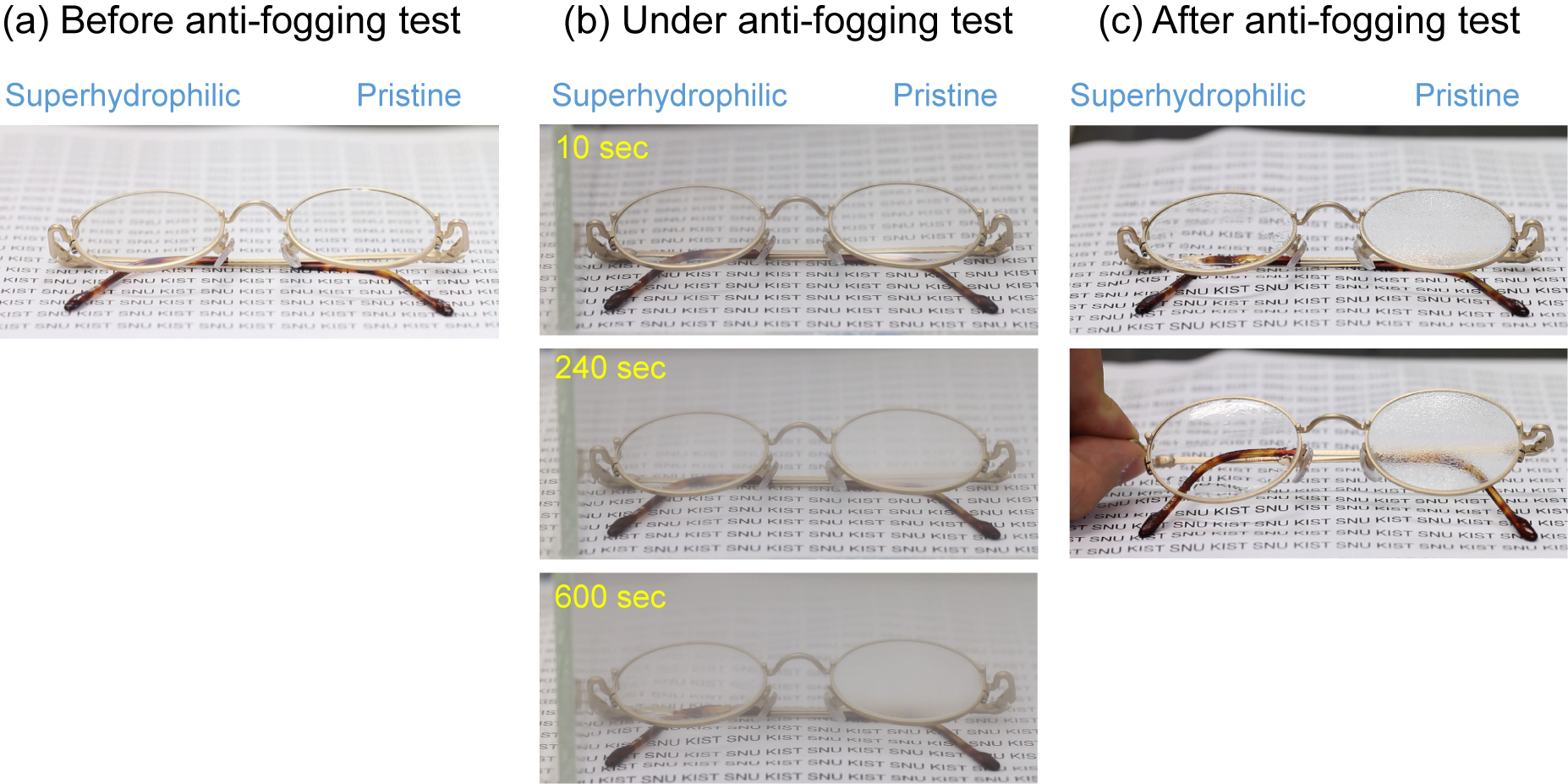


**Figure S4**. Optical images of superhydrophilic eye glasses taken (a) before, (b) under, and (c) after anti-fogging or condensation test: total test duration was 10 min under supersaturation conditions. The superhydrophilic glass shows a very thin water layer on the glass lens surface compared with the pristine glass after the anti-fogging test, as shown in the tilted images (bottom) in (c). The numbers in (b) are the durations of the tests.


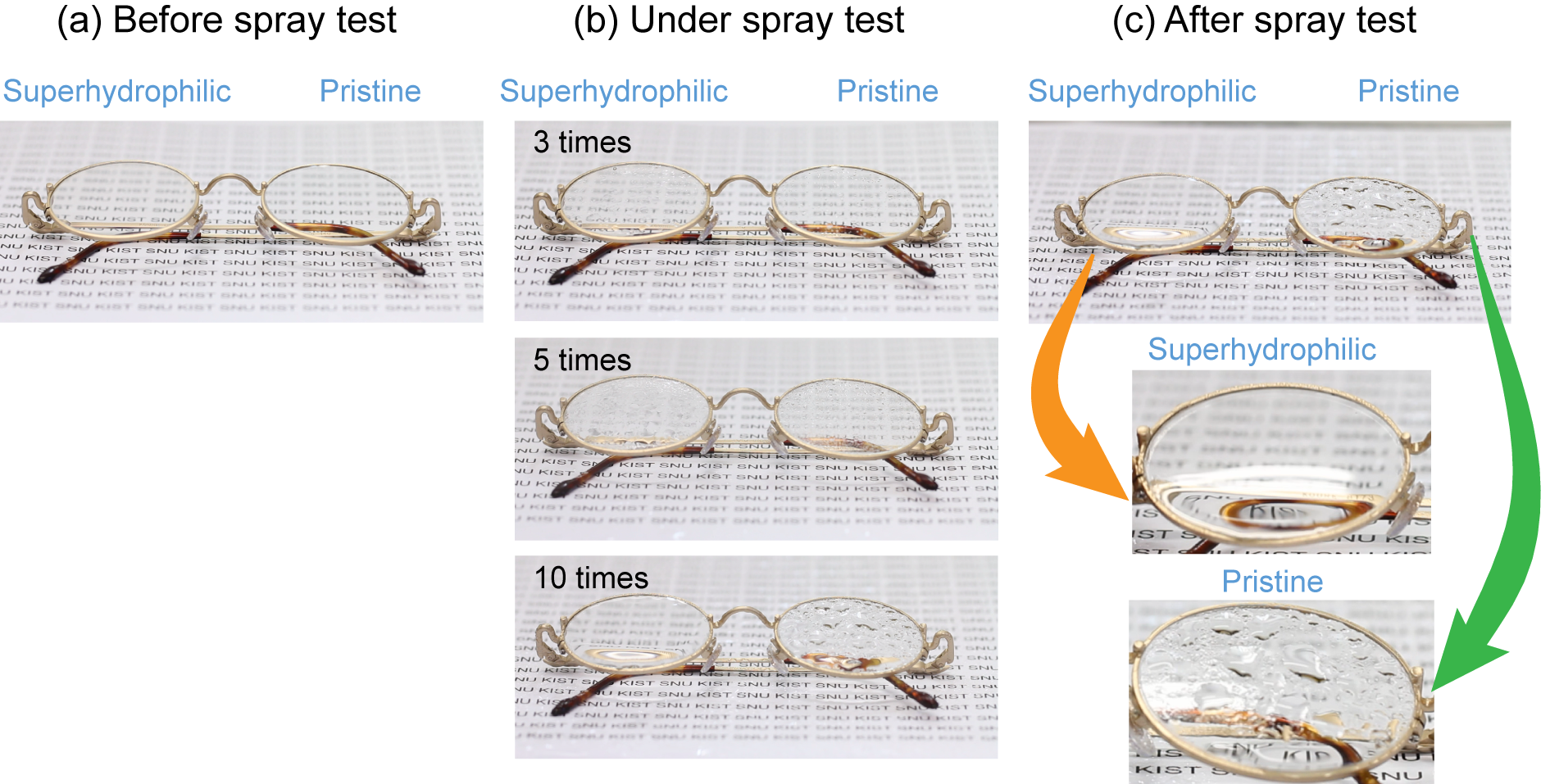


**Figure S5**. Optical images of superhydrophilic eye glasses taken (a) before, (b) under, and (c) after water spray. The total number of spraying cycles is 10. The superhydrophilic glass shows a water layer on the glass lens surface, whereas the pristine glass shows water droplets.
